# Genetic diversity of coronaviruses in *Miniopterus fuliginosus* bats

**DOI:** 10.1007/s11427-016-5039-0

**Published:** 2016-04-28

**Authors:** Jiang Du, Li Yang, Xianwen Ren, Junpeng Zhang, Jie Dong, Lilian Sun, Yafang Zhu, Fan Yang, Shuyi Zhang, Zhiqiang Wu, Qi Jin

**Affiliations:** 1 MOH Key Laboratory of Systems Biology of Pathogens, Institute of Pathogen Biology, Chinese Academy of Medical Sciences & Peking Union Medical College, Beijing, 100176 China; 2 Collaborative Innovation Center for Diagnosis and Treatment of Infectious Diseases, Hangzhou, 310003 China; 3 College of Animal Science and Veterinary Medicine, Shenyang Agricultural University, Shenyang, 110866 China; 4 State Key Laboratory of Estuarine and Coastal Research, Institute of Estuarine and Coastal Research, East China Normal University, Shanghai, 200062 China

**Keywords:** coronavirus, *Miniopterus fuliginosus*, bat, co-infection, recombination

## Abstract

Coronaviruses, such as severe acute respiratory syndrome coronavirus and Middle East respiratory syndrome coronavirus, pose significant public health threats. Bats have been suggested to act as natural reservoirs for both these viruses, and periodic monitoring of coronaviruses in bats may thus provide important clues about emergent infectious viruses. The Eastern bent-wing bat *Miniopterus fuliginosus* is distributed extensively throughout China. We therefore analyzed the genetic diversity of coronaviruses in samples of *M. fuliginosus* collected from nine Chinese provinces during 2011–2013. The only coronavirus genus found was *Alphacoronavirus*. We established six complete and five partial genomic sequences of alphacoronaviruses, which revealed that they could be divided into two distinct lineages, with close relationships to coronaviruses in *Miniopterus magnater* and *Miniopterus pusillus*. Recombination was confirmed by detecting putative breakpoints of Lineage 1 coronaviruses in *M. fuliginosus* and *M. pusillus* (Wu et al., 2015), which supported the results of topological and phylogenetic analyses. The established alphacoronavirus genome sequences showed high similarity to other alphacoronaviruses found in other *Miniopterus* species, suggesting that their transmission in different *Miniopterus* species may provide opportunities for recombination with different alphacoronaviruses. The genetic information for these novel alphacoronaviruses will improve our understanding of the evolution and genetic diversity of coronaviruses, with potentially important implications for the transmission of human diseases.

## INTRODUCTION

Coronaviruses (CoVs; order *Nidovirales*, family *Coronaviridae*, subfamily *Coronavirinae*) are enveloped RNA viruses with unusually large, positive-stranded RNA genomes of 26–32 kb (Lai, 2001). The viral genome contains five major open reading frames (ORFs) that encode the replicase polyproteins (ORF1a and ORF1b), spike (S), envelope (E), and membrane (M) glycoproteins, and the nucleocapsid protein (N) (Gonzalez et al., 2003; Holmes and Enjuanes, 2003). According to a proposal submitted to the International Committee on the Taxonomy of Viruses, CoVs can be classified into four genera, *Alphacoronavirus*, *Betacoronavirus*, *Gammacoronavirus*, and *Deltacoronavirus*, which replace the traditional CoV groups 1, 2, and 3 (King et al., 2011; Woo et al., 2009, 2012). CoVs are known to cause upper and lower respiratory diseases, gastroenteritis, and central nervous system infections in a number of avian and mammalian hosts, including humans (Weiss and Navas-Martin, 2005). Bats have been increasingly recognized as important natural reservoirs for CoVs. In particular, previously unknown CoVs related to severe human pathogens, such as severe acute respiratory syndrome (SARS) CoV (Li et al., 2005) and Middle East respiratory syndrome CoV (van Boheemen et al., 2012), were discovered in bats from China and other countries, with consequent recent increases in research into the biodiversity and genomics of CoVs in different bat species.

The diversity of CoVs arises from the infidelity of RNA-dependent RNA polymerase (RdRp), the high frequency of recombination, and the large genomes of CoVs (Woo, 2009). These factors have generated diverse strains and genotypes of the CoV lineage, and have given rise to new lineages able to adapt to new hosts. These new lineages have occasionally caused major zoonotic outbreaks with disastrous consequences (Woo, 2006).

A previous study reported the detection of several novel bat CoVs (BtCoVs) in *Miniopterus magnater* and *Miniopterus pusillus* from Hong Kong (Chu et al., 2008), and in *Miniopterus fuliginosus* from Japan (Shirato et al., 2012). However, despite being the most extensively distributed *Miniopterus* species in China, the CoVs harbored by *M. fuliginosus* (the Eastern bent-wing bat) have not been systematically studied. *M. fuliginosus* are known to migrate long distances and typically roost with large numbers of bats from different genera, including *Rhinolophus*, *Hipposideros*, and *Myotis* (Cui et al., 2007; Miller-Butterworth et al., 2003), which habits may facilitate viral exchange between different bat species. Furthermore, our understanding of the diversity of CoVs in the genus *Miniopterus* remains limited. We therefore launched a survey to determine the dynamics and prevalence of CoVs in *M. fuliginosus* living in different geographical regions. In the current study, we explored the genetic diversity of CoVs in *M. fuliginosus* in China by analyzing 194 bat samples collected from nine Chinese provinces during 2011–2013.

## RESULTS

### Bat surveillance and identification of CoVs

A total of 194 *M. fuliginosus* bats were captured in nine provinces of China from October 2010 to October 2013, and pharyngeal and anal swabs were collected (Figure 1). All sampling sites were in or close to human gathering places. Only the anal swab samples harbored CoVs according to single-strain screening with conserved primers, and the positivity rates for each province are shown in Figure 1. Sequence analysis of the PCR amplicons identified alpha-CoV-positive bats in six provinces (Guangdong, Hubei, Fujian, Henan, Anhui, and Jiangxi), but no other CoV genera were found. Interestingly, co-infections with different CoVs were detected in two *M. fuliginosus* anal specimens; one from Guangdong and one from Henan.

We selected samples positive for CoVs that were representative of each province for genomic sequencing and established the complete genomic sequences of six alpha-CoVs: BtMf-AlphaCoV/Guangdong2012 (GD), BtMf-AlphaCoV/Hubei2013 (HB), BtMf-Alpha CoV/Fujian2012 (FJ), BtMf-AlphaCoV/Henan2013 (HN), BtMf-AlphaCoV/

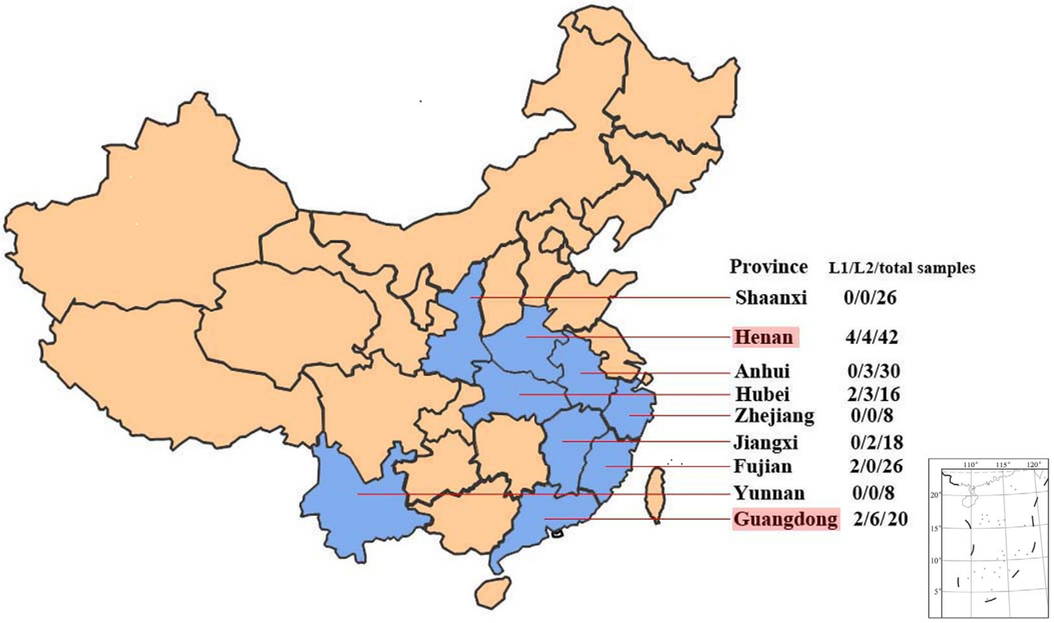

**Figure 1** The nine provinces (indicated in blue) in China, where bats were captured, and samples were collected. The numbers on the right indicate the numbers of samples positive for Lineage 1 (L1) and Lineage 2 (L2) and the total number of samples collected in each province. The red shading on Guangdong and Henan indicate the regions where co-infections of two lineages were detected.

Anhui2011 (AH), and BtMf-AlphaCoV/Jiangxi2012 (JX). We also established partial genomic sequences of five other alpha-CoVs: BtMf-AlphaCoV/Guangdong2012-a (GD-a), BtMf-AlphaCoV/Guangdong2012-b (GD-b), BtMf-AlphaCoV/ Hubei2013-a (HB-a), BtMf-AlphaCoV/Henan2013-a (HN-a), and BtMf-AlphaCoV/Henan2013-b (HN-b). The GD and GD-b sequences were identified in the same sample from Guangdong, and the HN and HN-b sequences were identified in the same sample from Henan.

## Genomic sequences

The sizes of the BtCoVs GD, HB, FJ, HN, AH, and JX genomes, excluding the 3′ poly(A) tails, were 28,748, 28,745, 28,755, 28,725, 28,300, and 28,301 nt, respectively, with G+C contents of 41.8%, 41.85%, 41.87%, 41.98%, 38.17%, and 38.19%, respectively. The genomic organization of these CoVs was similar to that of other alpha-CoVs (Table 1). The main difference among genomes was in ORF7, which was present in GD, HB, FJ, and HN, but absent in AH and JX. We then compared the complete genomes (Table 2). The full-length genomic sequences of HB, FJ, and HN showed 91.9%–97.0% nt identities with each another, and lower identity with the GD genome (82.1%–85.7%). In contrast, AH and JX exhibited 96.2% overall nt identity with each other, and lower identities with the other four genomes (68.0%–68.8%). The sizes of the 5′ untranslated regions of GD, HB, FJ, HN, AH, and JX were 270, 269, 268, 268, 272, and 273 nt, respectively. The core sequences of the leader transcription regulatory sequence (TRS; 5′-CUAAAC-3′) were identified in the 5′ untranslated sequences (Table 3). The TRSs of ORF3 and the *E* genes in AH and JX differed from those of the other four CoVs. The TRS of ORF7 in FJ and GD (CUGAAU) differed by 1 nt from that in HB and HN (CUGAAC). Apart from ORF3, E, and ORF7, the TRSs for the other ORFs were predicted in these six CoV genome sequences.

ORF1ab occupied approximately 70% of the genome, and consisted of ORF1a and ORF1b, encoding viral polyprotein 1a (pp1a) and pp1b, respectively. Putative features responsible for ribosomal frame shifting, e.g. the “slippage sequence” (5′-UUUAAAC-3′), were predicted in the genomes. ORF1a of AH and JX shared 98.5% aa identity, but lower (63.0%–63.8%) aa identity with the other four CoVs, while the ORF1a sequences of HB, FJ, and HN showed 99.2%–99.5% aa identity, but lower (87.5%–87.6%) aa identity with GD. The ORF1b sequences exhibited the same

**Table 1** Predicted ORFs in the genomes of bat CoVs[a)]

| ORFs | GD | | HB | | FJ | | HN | | AH | | JX | |
|---|---|---|---|---|---|---|---|---|---|---|---|---|
| | Position | Length (nt) | Position | Length (nt) | Position | Length (nt) | Position | Length (nt) | Position | Length (nt) | Position | Length (nt) |
| ORF1a | 271–12,966 | 12,693 | 270–12,944 | 12,672 | 269–12,943 | 12,672 | 269–12,943 | 12,672 | 273–13,076 | 12,801 | 274–13,077 | 12,801 |
| ORF1b | 12,936–20,960 | 8,022 | 12,914–20,938 | 8,022 | 12,913–20,937 | 8,022 | 12,913–20,937 | 8,022 | 13,046–21,067 | 8,019 | 13,047–21,068 | 8,019 |
| NSP1 | 271–600 | 330 | 270–599 | 330 | 269–598 | 330 | 269–598 | 330 | 273–599 | 327 | 274–600 | 327 |
| NSP2 | 601–2,943 | 2,343 | 600–2,942 | 2,343 | 599–2,941 | 2,343 | 599–2,941 | 2,343 | 600–2,951 | 2,352 | 601–2,952 | 2,352 |
| NSP3 | 2,944–8,175 | 5,232 | 2,943–8,153 | 5,211 | 2,942–8,152 | 5,211 | 2,942–8,152 | 5,211 | 2,952–8,288 | 5,337 | 2,953–8,289 | 5,337 |
| NSP4 | 8,176–9,600 | 1,425 | 8,154–9,578 | 1,425 | 8,153–9,577 | 1,425 | 8,153–9,577 | 1,425 | 8,289–9,710 | 1,422 | 8,290–9,711 | 1,422 |
| NSP5 | 9,601–10,506 | 906 | 9,579–10,484 | 906 | 9,578–10,483 | 906 | 9,578–10,483 | 906 | 9,711–10,616 | 906 | 9,712–10,617 | 906 |
| NSP6 | 10,507–11,343 | 837 | 10,485–11,321 | 837 | 10,484–11,320 | 837 | 10,484–11,320 | 837 | 10,617–11,453 | 837 | 10,618–11,454 | 837 |
| NSP7 | 11,344–11,592 | 249 | 11,322–11,570 | 249 | 11,321–11,569 | 249 | 11,321–11,569 | 249 | 11,454–11,702 | 249 | 11,455–11,703 | 249 |
| NSP8 | 11,593–12,174 | 582 | 11,571–12,152 | 582 | 11,570–12,151 | 582 | 11,570–12,151 | 582 | 11,703–12,284 | 582 | 11,704–12,285 | 582 |
| NSP9 | 12,175–12,504 | 330 | 12,153–12,482 | 330 | 12,152–12,481 | 330 | 12,152–12,481 | 330 | 12,285–12,614 | 330 | 12,286–12,615 | 330 |
| NSP10 | 12,505–12,912 | 408 | 12,483–12,890 | 408 | 12,482–12,889 | 408 | 12,482–12,889 | 408 | 12,615–13,022 | 408 | 12,616–13,023 | 408 |
| NSP11 | 12,913–12,966 | 54 | 12,891–12,944 | 54 | 12,890–12,943 | 54 | 12,890–12,943 | 54 | 13,023–13,076 | 54 | 13,024–13,077 | 54 |
| NSP12 | 12,913–15,692 | 2,781 | 12,891–15,670 | 2,781 | 12,890–15,669 | 2,781 | 12,890–15,669 | 2,781 | 13,023–15,802 | 2,781 | 13,024–15,803 | 2,781 |
| NSP13 | 15,693–17,483 | 1,791 | 15,671–17,461 | 1,791 | 15,670–17,460 | 1,791 | 15,670–17,460 | 1,791 | 15,803–17,584 | 1,782 | 15,804–17,585 | 1,782 |
| NSP14 | 17,484–19,040 | 1,557 | 17,462–19,018 | 1,557 | 17,461–19,017 | 1,557 | 17,461–19,017 | 1,557 | 17,585–19,147 | 1,563 | 17,586–19,145 | 1,560 |
| NSP15 | 19,041–20,057 | 1,017 | 19,019–20,035 | 1,017 | 19,018–20,034 | 1,017 | 19,018–20,034 | 1,017 | 19,148–20,164 | 1,017 | 19,146–20,165 | 1,020 |
| NSP16 | 20,058–20,960 | 900 | 20,036–20,938 | 900 | 20,035–20,937 | 900 | 20,035–20,934 | 900 | 20,165–21,067 | 900 | 20,166–21,068 | 900 |
| S | 20,962–25,098 | 4,134 | 20,935–25,059 | 4,122 | 20,939–25,075 | 4,134 | 20,939–25,075 | 4,134 | 21,069–25,196 | 4,125 | 21,070–25,200 | 4,128 |
| ORF3 | 25,098–25,766 | 666 | 25,059–25,727 | 666 | 25,075–25,743 | 666 | 25,075–25,743 | 666 | 25,196–25,855 | 657 | 25,200–25,859 | 657 |
| E | 25,750–25,974 | 222 | 25,711–25,935 | 222 | 25,727–25,951 | 222 | 25,727–25,951 | 222 | 25,849–26,073 | 222 | 25,853–26,077 | 222 |
| M | 25,984–26,742 | 756 | 25,945–26,709 | 762 | 25,961–26,719 | 756 | 25,961–26,719 | 756 | 26,080–26,841 | 759 | 26,084–26,842 | 756 |
| N | 26,791–28,059 | 1,266 | 26,758–28,026 | 1,266 | 26,768–28,036 | 1,266 | 26,768–28,036 | 1,266 | 26,862–28,031 | 1,167 | 26,863–28,032 | 1,167 |
| ORF7a | 27,809–27,979 | 168 | 27,776–28,522 | 744 | 27,786–28,532 | 744 | 27,786–28,505 | 717 | | | | |
| ORF7b | 28,034–28,528 | 492 | | | | | | | | | | |

a) BtMf-AlphaCoV/Guangdong2012 (GD), BtMf-AlphaCoV/Hubei2013 (HB), BtMf-AlphaCoV/Fujian2012 (FJ), BtMf-AlphaCoV/Henan2013 (HN), BtMf-AlphaCoV/Anhui2011 (AH), and BtMf-AlphaCoV/Jiangxi2012 (JX).

**Table 2** Percent nucleotide identity between whole genomes and percent amino acid similarities between viral protein sequences in bat CoVs[a)]

| Nucleotide or protein | Virus | Lineage 1 | | | | Lineage 2 | | |
|---|---|---|---|---|---|---|---|---|
| | | GD | HB | FJ | HN | AH | JX | 1A |
| Genome | HKU8 | 91.8 | 86.1 | 82.2 | 81.6 | 67.7 | 67.6 | 67.7 |
| | GD | – | 82.1 | 85.4 | 85.7 | 68.6 | 68.5 | 68.5 |
| | HB | – | – | 92.8 | 91.9 | 68.1 | 68.0 | 68.0 |
| | FJ | – | – | – | 97.0 | 68.8 | 68.8 | 68.8 |
| | HN | – | – | – | – | 68.7 | 68.7 | 68.6 |
| | AH | – | – | – | – | – | 96.2 | 96.2 |
| | JX | – | – | – | – | – | – | 96.0 |
| ORF1a | HKU8 | 99.0 | 87.2 | 87.1 | 87.3 | 63.4 | 63.4 | 63.0 |
| | GD | – | 87.6 | 87.5 | 87.6 | 63.5 | 63.5 | 63.2 |
| | HB | – | – | 99.2 | 99.5 | 63.6 | 63.7 | 63.3 |
| | FJ | – | – | – | 99.3 | 63.7 | 63.7 | 63.3 |
| | HN | – | – | – | – | 63.6 | 63.6 | 63.2 |
| | AH | – | – | – | – | – | 98.5 | 97.7 |
| | JX | – | – | – | – | – | – | 98.4 |
| ORF1b | HKU8 | 99.6 | 98.2 | 98.2 | 98.2 | 87.9 | 87.7 | 87.4 |
| | GD | – | 98.3 | 98.2 | 98.3 | 88.0 | 87.8 | 87.5 |
| | HB | – | – | 99.8 | 99.8 | 88.0 | 87.8 | 87.5 |
| | FJ | – | – | – | 99.9 | 87.9 | 87.7 | 87.4 |
| | HN | – | – | – | – | 87.9 | 87.7 | 87.4 |
| | AH | – | – | – | – | – | 99.8 | 99.4 |
| | JX | – | – | – | – | – | – | 99.3 |
| RDRP | HKU8 | 99.8 | 97.1 | 97.1 | 97.0 | 90.1 | 89.9 | 90.0 |
| | GD | – | 97.1 | 97.1 | 97.0 | 90.1 | 89.9 | 90.0 |
| | HB | – | – | 100.0 | 99.9 | 90.2 | 90.0 | 90.1 |
| | FJ | – | – | – | 99.9 | 90.2 | 90.0 | 90.1 |
| | HN | – | – | – | – | 90.1 | 89.9 | 90.0 |
| | AH | – | – | – | – | – | 99.8 | 99.9 |
| | JX | – | – | – | – | – | – | 99.7 |
| S | HKU8 | 52.9 | 95.7 | 53.5 | 53.5 | 49.0 | 48.4 | 49.1 |
| | GD | – | 52.5 | 87.8 | 87.5 | 61.0 | 60.7 | 60.6 |
| | HB | – | – | 52.7 | 52.8 | 49.1 | 48.6 | 49.2 |
| | FJ | – | – | – | 98.0 | 60.7 | 59.6 | 60.5 |
| | HN | – | – | – | – | 60.9 | 59.6 | 60.6 |
| | AH | – | – | – | – | – | 93.2 | 93.2 |
| | JX | – | – | – | – | – | – | 91.6 |
| ORF3 | HKU8 | 97.8 | 98.2 | 97.8 | 97.3 | 46.3 | 46.3 | 46.3 |
| | GD | – | 99.6 | 99.1 | 98.7 | 46.3 | 46.3 | 46.3 |
| | HB | – | – | 99.6 | 99.1 | 46.3 | 46.3 | 46.3 |
| | FJ | – | – | – | 99.6 | 46.3 | 46.3 | 46.3 |
| | HN | – | – | – | – | 46.3 | 46.3 | 46.3 |
| | AH | – | – | – | – | – | 99.5 | 99.1 |
| | JX | – | – | – | – | – | – | 98.6 |
| E | HKU8 | 98.7 | 98.7 | 98.7 | 98.7 | 70.7 | 70.7 | 70.7 |
| | GD | – | 100.0 | 100.0 | 100.0 | 70.7 | 70.7 | 70.7 |
| | HB | – | – | 100.0 | 100.0 | 70.7 | 70.7 | 70.7 |
| | FJ | – | – | – | 100.0 | 70.7 | 70.7 | 70.7 |
| | HN | – | – | – | – | 70.7 | 70.7 | 70.7 |
| | AH | – | – | – | – | – | 100.0 | 100.0 |
| | JX | – | – | – | – | – | – | 100.0 |
| M | HKU8 | 85.6 | 85.3 | 85.6 | 85.6 | 72.2 | 72.5 | 73.0 |
| | GD | – | 93.7 | 99.6 | 99.2 | 73.3 | 73.6 | 73.1 |
| | HB | – | – | 93.7 | 93.7 | 71.5 | 71.8 | 72.9 |
| | FJ | – | – | – | 99.6 | 73.3 | 73.6 | 73.1 |
| | HN | – | – | – | – | 72.9 | 73.2 | 73.1 |
| | AH | – | – | – | – | – | 99.6 | 93.3 |
| | JX | – | – | – | – | – | – | 93.7 |

(*To be continued on the next page*)

(*Continued*)

| Nucleotide or protein | Virus | Lineage 1 | | | | Lineage 2 | | |
|---|---|---|---|---|---|---|---|---|
| | | GD | HB | FJ | HN | AH | JX | 1A |
| N | HKU8 | 93.9 | 88.9 | 88.2 | 87.9 | 64.3 | 64.1 | 64.3 |
| | GD | – | 91.5 | 90.3 | 90.1 | 63.8 | 63.6 | 63.8 |
| | HB | – | – | 98.6 | 97.9 | 65.9 | 65.6 | 65.6 |
| | FJ | – | – | – | 98.3 | 66.1 | 65.9 | 65.9 |
| | HN | – | – | – | – | 65.6 | 65.4 | 65.4 |
| | AH | – | – | – | – | – | 99.7 | 98.7 |
| | JX | – | – | – | – | – | – | 99.0 |
| ORF7 | HKU8 | 61.0 | 84.7 | 84.8 | 59.0 | | | |
| | GD | – | 61.3 | 61.0 | 96.5 | | | |
| | HB | – | – | 97.9 | 61.7 | | | |
| | FJ | – | – | – | 63.0 | | | |
| | HN | – | – | – | – | | | |

a) BtMf-AlphaCoV/Guangdong2012 (GD), BtMf-AlphaCoV/Hubei2013 (HB), BtMf-AlphaCoV/Fujian2012 (FJ), BtMf-AlphaCoV/Henan2013 (HN), BtMf-AlphaCoV/Anhui2011 (AH), and BtMf-AlphaCoV/Jiangxi2012 (JX), HKU8, and 1A.

tendencies in terms of sequence similarities. Based on a previous analysis, the pp1a and pp1b proteins were predicted to be cleaved by virus proteases to produce a total of 16 nonstructural proteins (NSPs) (Chen et al., 2003). ORF1ab in GD, HB, FJ, HN, AH, and JX CoVs contained functional units typical of CoVs (Table 1), including *RdRps* in the NSP12 region. RdRp is a highly conserved CoV protein that is frequently used for phylogenetic comparisons. Six CoV genome sequences had *RdRps* genes of the same size (2781 nt). aa-sequence identity analyses of the RdRp proteins (Table 2) suggested that the six alpha-CoVs could be divided into two lineages: Lineage 1, including GD, HB, FJ, and HN, which shared 97%–100% aa identity, and Lineage 2, including AH and JX, which were closely related to each other (99.8% aa identity) and showed lower (89.9%–90.2%) aa identity with Lineage 1 CoVs.

Comparison of the aa sequences of the seven conserved replicase domains or NSPs (ADP-ribose-1′-phosphatase, NSP5 ($3CL^{pro}$), NSP12 (RdRp), NSP13 (Hel), NSP14 (3′→5′ exonuclease; (guanine-N7)-methyltransferase), NSP15 (nidoviraluridylate-specific endoribonuclease), and NSP16 (2′-O-ribose methyltransferase) for CoV species demarcation (de Groot, 2011) showed that Lineage 1 and Lineage 2 possessed <90% aa-sequence identity with each other, and BtCoV-HKU8 showed high aa identities (87.9%–93.9%) in terms of N protein with other Lineage 1 CoVs (GD, FJ, HB, HN). The N protein aa identities between the Lineage 2 CoVs AH, JX and BtCoV-1A, BtCoV-1B were 98.7%–99% and 91.6%–91.9%, respectively, indicating that Lineage 1 and Lineage 2 represented different species of *Alphacoronavirus*.

The most striking differences among CoVs were observed in the S protein sequence. The *S* gene sequence had five nts (AAAAU) inserted between the TRS and AUG in all CoVs except HB CoV (Table 3). Interestingly, the S protein (1,378 aa) was the same size in all members of Lineage 1, except HB (1,374 aa). However, the HB S protein shared only about 52.5%–52.8% aa identities with the S proteins of other Lineage 1 CoVs. Among the other Lineage 1 CoVs, the S proteins of FJ and HN were 98.0% identical, but they shared only 87.5% and 87.8% aa identity, respectively, with GD. In Lineage 2, AH and JX S proteins were 93.2% identical. Notably, the S proteins of GD, FJ, and HN in Lineage 1 appeared to be more closely related to the S proteins of Lineage 2 CoVs (59.6%–61.0%) than to the S protein of HB (52.5%–52.8%). Inter-ProScan analysis predicted that all six CoVs included type I membrane glycoproteins, where most of the protein (prior to residues 1318/1319/1322) was exposed on the outside of the viral capsule, and the C terminus comprised a transmembrane domain (residues 1319/1320/1323–1341/1342/1345), followed by the internal region in the virion, which was rich in cysteine residues. The S protein responsible for virus entry was divided into two domains; the S1 domain involved in receptor binding and the S2 domain for cellular membrane fusion. The putative S1 region was located at residues 229–741 for HB; 227–739 for GD and AH, 228–740 for JX, and 224–739 for FJ and HN. The diversity of S proteins was mainly within the S1 domain. HB S1 showed 93.3% aa identity with BtCoV-HKU8 and 39.6%–41.5% with other Lineage 1 and Lineage 2 CoVs. AH shared high aa identities with Lineage 2 CoVs in the S1 region (86.8%–93.7%), and GD had 85.1%–85.7% aa identities with FJ and HN. Analysis of the aa identities of the S1 region were consistent with the phylogenetic trees for the whole S region (Figure 2). S2 included two putative heptad repeat regions, important for membrane fusion and viral entry (Bosch et al., 2003), located at residues 977–1122 and 1264–1320 in GD, FJ, and HN, 975–1120 and 1260–1316 in HB, and 973/974–1122/1123 and 1252/1253–1311/1312 in AH and JX.

ORF3, which encoded putative 222-aa and 219-aa proteins in Lineage 1 and Lineage 2 CoVs, respectively, was located between the S and E sequences in all six genomes.

**Table 3** Transcription regulatory sequences (TRSs) for six bat CoVs[a)]

| ORF TRS | CoV | TRS sequence | Nucleotide position |
|---|---|---|---|
| Leader TRS | GD | CUCAA CUAAAC GAAAU | 69 |
| | HB | CUCAA CUAAAC GAAAU | 68 |
| | FJ | CUCAA CUAAAC GAAAU | 67 |
| | HN | CUCAA CUAAAC GAAAU | 67 |
| | AH | CUCAA CUAAAC GAAAU | 68 |
| | JX | CUCAA CUAAAC GAAAU | 69 |
| S | GD | UUCAA CUAAAU AAAAUG | 20,953 |
| | HB | UUCAA CUAAAU G | 20,931 |
| | FJ | UUCAA CUAAAU AAAAUG | 20,930 |
| | HN | UUCAA CUAAAU AAAAUG | 20,930 |
| | AH | UUCAA CUAAAU AAAAUG | 21,060 |
| | JX | UUCAA CUAAAU AAAAUG | 21,061 |
| ORF3 | GD | UACAA CAAUAC GAAGUN$_{21}$AUG | 25,066 |
| | HB | UACAA CAAUAC GAAGUN$_{21}$AUG | 25,027 |
| | FJ | UACAA CAAUAC GAAGUN$_{21}$AUG | 25,043 |
| | HN | UACAA CAAUAC GAAGUN$_{21}$AUG | 25,043 |
| | AH | UACAA CGUUAC GAAAUN$_{21}$AUG | 25,164 |
| | JX | UACAA CGUUAC GAAAUN$_{21}$AUG | 25,168 |
| E | GD | UACAA CUCUAC GAAGAUG | 25,740 |
| | HB | UACAA CUCUAC GAAGAUG | 25,701 |
| | FJ | UACAA CUCUAC GAAGAUG | 25,717 |
| | HN | UACAA CUCUAC GAAGAUG | 25,717 |
| | AH | UUCAA CUACAC GAAGAUG | 25,839 |
| | JX | UUCAA CUACAC GAAGAUG | 25,843 |
| M | GD | GAUGU CUAAAC GAACAAAAUG | 25,971 |
| | HB | GAUGU CUAAAC GAACAAAAUG | 25,932 |
| | FJ | GAUGU CUAAAC GAACAAAAUG | 25,948 |
| | HN | GAUGU CUAAAC GAACAAAAUG | 25,948 |
| | AH | AAUGU CUAAAC GAGAAUG | 26,070 |
| | JX | AAUGU CUAAAC GAGAAUG | 26,074 |
| N | GD | AUAAA CUAAAC AAGUGN$_{36}$AUG | 26,744 |
| | HB | AUAAA CUAAAC AAGUGN$_{36}$AUG | 26,711 |
| | FJ | AUAAA CUAAAC AAGUGN$_{36}$AUG | 26,721 |
| | HN | AUAAA CUAAAC AAGUGN$_{36}$AUG | 26,721 |
| | AH | UUAAA CUAAAC AAGAAN$_{8}$AUG | 26,843 |
| | JX | UUAAA CUAAAC AAGAAN$_{8}$AUG | 26,844 |
| ORF7 | GD | GAUUG CUGAAU UGCUAN$_{88}$AUG | 27,710 |
| | HB | AAUUG CUGAAC UGAUUN$_{88}$AUG | 27,677 |
| | FJ | AAUUG CUGAAU UGAUUN$_{88}$AUG | 27,687 |
| | HN | AAUUG CUGAAC UGAUCN$_{88}$AUG | 27,687 |

a) For putative ORFs, we aligned the TRS that preceded the start codon AUG with the leader TRS. The core sequence is indicated in a box. The start codons of genes are in bold type.

The aa sequences of ORF3 were highly conserved within Lineages 1 and 2 (98.7%–99.6% and 99.5%, respectively), but varied between lineages (46.3%). Among the CoV proteins, ORF3 showed the greatest inter-lineage diversity. Multiple transmembrane motifs were predicted in ORF3 proteins, suggesting that they might be surface proteins. TMHMM analysis showed that Lineage 1 CoVs harbored three putative transmembrane domains in ORF3 (aa residues 36–58, 70–92, and 96–113), while Lineage 2 CoVs harbored only two putative transmembrane domains (aa residues 37–59 and 71–93).

The E, M, and N proteins were highly conserved within CoVs of the same lineage (>90% identity) and were diverse between lineages (63.6%–73.6%). ORF7 was located at the 3′ end of the Lineage 1 virus genome, and overlapped with the *N* gene. ORF7 encoded a putative NSP of 239–248 aa residues in FJ, HN, and HB. Interestingly, ORF7 in GD possessed two small ORFs, encoding putative proteins of 56 and 164 aa residues, respectively (Table 1).

## Phylogenetic analyses

We performed phylogenetic analyses based on the aa sequences of the RdRp, S, E, M, and N proteins of these BtCoVs, including the RdRp and S proteins in the five partial CoV sequences (GD-a, GD-b, HB-a, HN-a, and HN-b). Phylogenetic trees were constructed using MEGA5.0 software, based on the deduced aa sequences. Several reference CoV genome sequences were downloaded from GenBank and aligned with the fragments of the newly discovered CoVs (Figure 2). The results of the phylogenetic analyses were consistent with those of the sequence identity analyses, and confirmed that the newly identified alpha-CoVs could be divided into two lineages. The aa sequences of the RdRp, E, M, and N proteins in Lineage 1 viruses always clustered with BtCoV HKU8, found in *M. pusillus*. In contrast, phylogenetic analysis based on the S proteins showed a different tree structure, in which GD, FJ, and HN in Lineage 1 clustered together in a clade with Lineage 2 viruses, and HB and BtCoV HKU8 formed a relatively distant cluster, sharing 95.7% aa identity with each other and only 52.7%–53.5% identity with the other three Lineage 1 CoVs. Phylogenetic analysis of the S protein thus indicated that Lineage 1 CoVs could be further divided into two types: type I (HB and HKU8) and type II (FJ, HN, and GD). According to the phylogenetic trees, Lineage 2 viruses (AH, JX, GD-a, HB-a, and HN-a) always clustered with BtCoV 1A, found in *M. magnater* (>99.7% nt identity in RdRp and >91.4% aa in S protein), and GD-b and HN-b with BtCoV 1B, found in *M. pusillus* (98.7% aa identity with RdRp and about 92.0% with S protein). These tree branches were very short, reflecting the high sequence similarities.

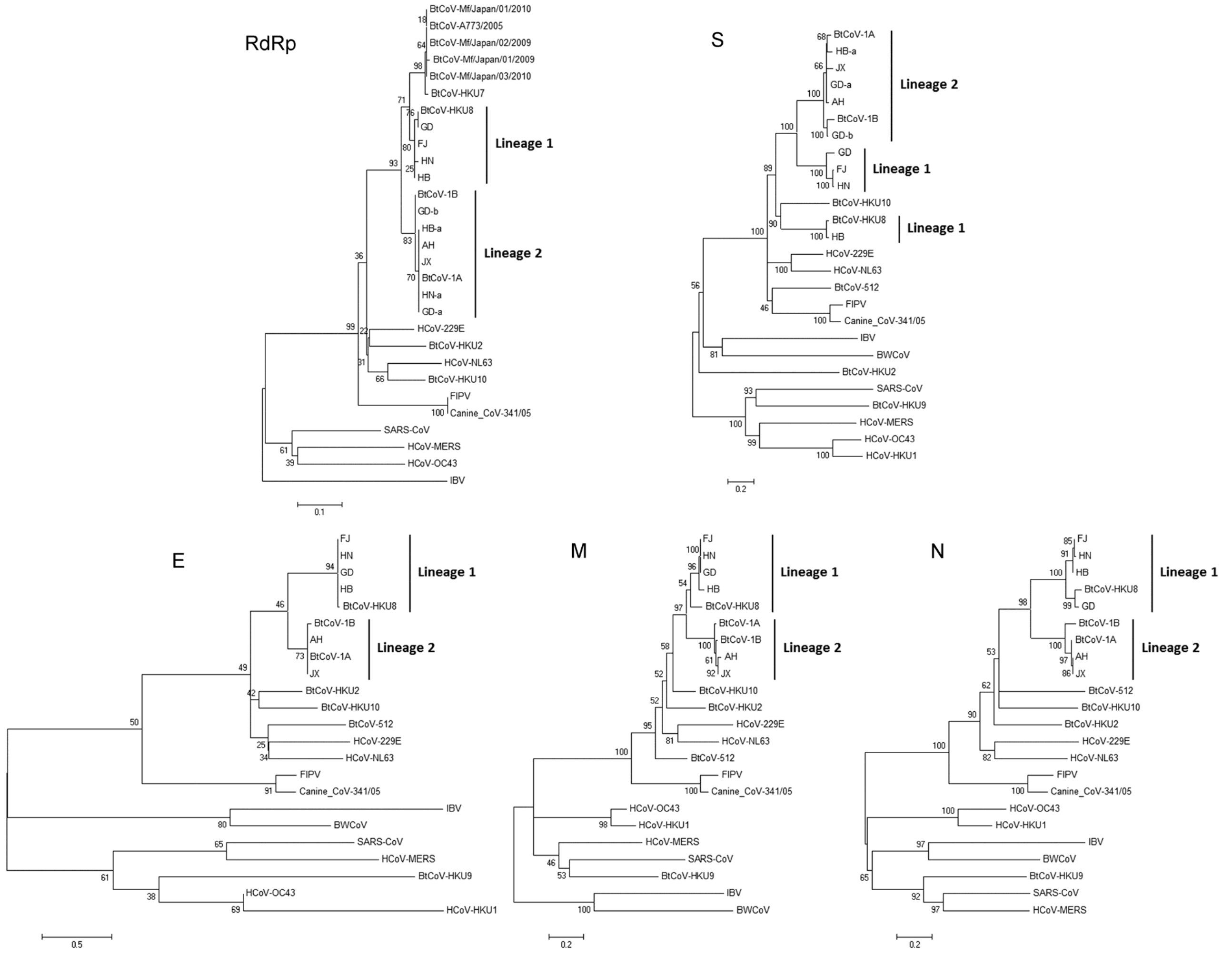

**Figure 2** Phylogenetic trees based on the amino acid sequences of the partial RNA-dependent RNA polymerase (RdRp; an 324-nt sequence fragment corresponding to positions 14828–15151 in bat coronavirus (BtCoV-HKU8; NC010438)), full-length spike (S), envelope (E), membrane (M), and nucleocapsid (N) proteins. The following CoVs and GenBank accession numbers were used: BtCoV-1A (NC010437), BtCoV-1B (NC010436), BtCoV-HKU7 (DQ249226), BtCoV-HKU2 (NC009988), BtCoV-HKU10 (NC018871), BtCoV-512 (NC009657), BtCoV-Mf/Japan/01/2009 (AB619638), BtCoV-Mf/Japan/02/2009 (AB619639), BtCoV-Mf/Japan/01/2010 (AB619640), BtCoV-Mf/Japan/03/2010 (AB619642), BtCoV-A773/2005 (DQ648835), Feline infectious peritonitis virus (FIPV; AY994055), Canine CoV-341/05 (EU856361), BtCoV-HKU9 (EF065513), severe acute respiratory syndrome coronavirus (SARS-CoV; NC004718), human CoV OC43 (HCoV-OC43; NC005147), HCoV-HKU1 (NC006577), HCoV-229E (NC002645), HCoV-NL63 (NC005831), Middle East respiratory syndrome coronavirus (HCoV-MERS; KF192507), avian infectious bronchitis virus (IBV; NC001451), beluga whale CoV SW1 (BWCoV; NC010646). Scale bar indicates genetic distance, estimated with a WAG+G model implemented in MEGA5 (www.megasoftware.net).

### Recombination analyses

Co-infection with different CoVs in the same bat may create opportunities for recombination, potentially resulting in the emergence of new viruses. Co-infections with different lineages in *M. fuliginosus* were detected in two anal specimens collected in Guangdong and Henan (Wu et al., 2015). Previous studies have shown that CoVs have a tendency to undergo RNA recombination (Herrewegh et al., 1998; Lai and Cavanagh, 1997; Lau et al., 2012b; Makino et al., 1986; Zeng et al., 2008). In this study, we found that recombinant events had occurred among the four Lineage 1 sequences (FJ, GD, HN, HB) and BtCoV HKU8. GD showed the highest degree of similarity to BtCoV HKU8 in the ORF1ab region with an aa identity >99% (Table 2). The ORF1ab region of GD may have originated from BtCoV HKU8 during a co-infection event in the same bat species. However, HB showed the highest degree of similarity to BtCoV HKU8 in the S region, with an aa identity of 95.7% (Table 2). The S region of HKU8 may be the parental sequence of the equivalent region in HB. Considering the diversity of the S region in Lineage 1 CoVs, we analyzed possible recombination events in Lineage 1 BtCoVs from different sites in China by detecting putative breakpoints and using SimPlot software (Wu et al., 2015). GARD analysis results were consistent with the bootscan analysis results, and three recombination breakpoints were found in the alignments of GD, HB, HN, FJ, and BtCoV HKU8 from *M. pusillus* (nt 20,930, nt 26,861, and nt 28,128, respectively) (Wu et al., 2015). The positions of the detected breakpoints corresponded to the areas of recombination.

## DISCUSSION

In this study, we detected and characterized alpha-CoVs carried by *M. fuliginosus* bats in China. *M. fuliginosus*-related alpha-CoVs were detected in six different provinces (Guangdong, Hubei, Fujian, Henan, Anhui, and Jiangxi), representing the middle, eastern, and southern parts of China. Based on genetic and phylogenetic analyses, these alpha-CoVs could be classified into two distinct lineages, Lineage 1 and Lineage 2. Lineage 1/Lineage 2 co-infections were detected in two specimens collected from Guangdong and Henan (Wu et al., 2015).

Lineage 1 and Lineage 2 CoVs showed high intra-lineage genomic similarities, except in the S region. This high similarity suggests each lineage shared a common ancestor. However, Lineage 1 genomes (GD, HB, FJ, and HN), isolated from Guangdong, Hubei, Fujian, and Henan provinces, presented marked differences in the S region, and phylogenetic analysis of S proteins showed that Lineage 1 CoVs formed two distinct clusters, comprising GD, FJ, and HN in one cluster, and HB in a relatively distant cluster. The same CoV in one bat species had thus evolved diverse S proteins in different provinces. Different environmental pressures, including food availability, climate, shelter, and predators, may have exerted different selection pressures on the CoVs in the same bat species in different locations, leading to the emergence of a novel S protein subtype in the same CoV isolated from different regions.

The S protein in CoV is responsible for receptor binding and host-species adaptation, and is one of the major determinants of specificity of host-species infection (Dveksler et al., 1991; Lau et al., 2005, 2007). The S protein gene therefore constitutes one of the most variable regions within the CoV genome. GD in *M. fuliginosus* and BtCoV HKU8 in *M. pusillus* showed a higher degree of genomic similarity than any of the other CoVs, except in the S region. Phylogenetic analysis of the S protein revealed that BtCoV HKU8 clustered with HB, rather than with GD; indeed the BtCoV HKU8 S protein exhibited higher identity with HB than the other three Lineage 1 CoVs, including GD. Phylogenetic analysis, similarity plots, bootscan analysis, and recombination-breakpoint analysis suggested that recombination occurred around the S region among BtCoV HKU8, GD, and HB (Wu et al., 2015), which may have facilitated adaptation of the virus to a new bat species, finally leading to interspecies transmission (Graham and Baric, 2010; Song et al., 2005). Furthermore, within the complete genome (including the S region), some of the established Lineage 2 CoVs (AH, JX, GD-a, HB-a, and HN-a) showed high similarity to BtCoV 1A found in *M. magnater*, while other Lineage 2 CoVs (GD-b and HN-b) showed high similarity to BtCoV 1B found in *M. pusillus*. Overall, bat migration and roosting habits provide opportunities for large numbers of bats to gather together (Cui et al., 2007; Woo et al., 2006a, 2006c; Woo, 2006), and could explain the mechanisms whereby *Miniopterus* acquires various viruses and transmits them to other bat species. In addition, our findings also suggested that the S protein had undergone varying degrees of modification in response to the evolutionary pressure of adapting to a new host.

Previous studies found that CoVs are particularly host-specific, though host-shifting has also been demonstrated (Jonassen et al., 2005; Lai, 1990; Liu et al., 2005; Rest and Mindell, 2003). A larger-scale study including different geographic regions will be necessary to confirm the phenomenon of host specificity. The results of the present study showed that a single bat species (*M. fuliginosus*) could harbor more than one species of CoV (Lineage 1 and 2 CoVs), and that one CoV could be found in different species of bats, indicating no strict association between BtCoVs and bat species. The availability of genomic-sequence data for CoVs from bat species from different locations will allow analysis of the relationships between these viruses and the geographic distribution of their hosts. Further characterization of novel CoVs revealed high genetic diversity across a large geographic distribution. Moreover, we found that the same species of bat from different geographic locations contained the same species of

CoV, but with distinct S proteins.

The novel genomes described in this study represent the first genomic data for CoVs in *M. fuliginosus* bats in China. The results also provide the first evidence for the high diversity of S proteins within a given CoV carried by the same bat species at different locations. This diversity most likely arose as a result of environmental pressures, migration abilities, and roosting behaviors (Lau et al., 2012a). Conversely, highly similar CoV genomes, including similar or diverse S regions, were found in different bat species from different regions, suggesting that recombination and interspecies transmission may occur among BtCoVs. Recombination may create opportunities for the emergence of new viruses that might drive CoV evolution (Vijaykrishna et al., 2007; Woo et al., 2006b). Previous studies demonstrated that SARS and a number of other new human diseases have emerged as a result of interspecies transmission of viruses carried by bats. The genetic features and host restriction of BtCoVs thus remain important subjects for global public health studies. Further studies and genomic analyses of CoVs from different *Miniopterus* species in different regions will contribute to a better understanding of the diversity and evolution of CoVs, and periodic studies could provide genetic clues regarding potential emergent infectious viruses.

## MATERIALS AND METHODS

### Ethics statement

The field studies did not involve endangered or protected species. Bats were treated according to the guidelines set out in the Regulations for the Administration of Laboratory Animals (Decree No. 2 of the State Science and Technology Commission of the People's Republic of China, 1988). The sampling procedures were approved by the Ethics Committee of the Institute of Pathogen Biology, Chinese Academy of Medical Sciences & Peking Union Medical College (Approval number: IPB EC20100415).

### Bat samples

Pharyngeal and anal swabs were collected from 194 captured *M. fuliginosus* bats from nine provinces in China. No specific permissions were required for these procedures at these locations. All bats trapped for this study were released back into their habitat after sample collection. The bat species was initially determined morphologically and subsequently confirmed by sequence analysis of mitochondrial cytochrome b DNA, as described previously (Tang et al., 2006). The samples were immersed in maintenance medium in virus-sampling tubes (Yocon, China), temporarily stored at –20°C, and then transported to the laboratory and stored at –80°C.

### RNA extraction and virus detection

Viral RNA was extracted from the pharyngeal and anal swab samples using a QIAamp viral RNA minikit (Qiagen, Germany). Reverse transcription was performed using a SuperScript III kit (Invitrogen, USA). CoV screening was performed by amplifying a 440-bp fragment of the *RdRp* gene of CoVs using conserved primers (5′-GGTTGGG-ACTATCCTAAGTGTGA-3′ and 5′-CCATCATCAGATA-GA-ATCATCATA-3′), as described previously (Lau et al., 2012a, 2012b). Polymerase chain reaction (PCR) products were gel purified using a QIAquick gel extraction kit (Qiagen). Both strands of the PCR products were sequenced twice with an ABI Prism 3700 DNA analyzer (Applied Biosystems, USA), using the two PCR primers. The sequences of the PCR products were compared with known CoV *RdRp* gene sequences in the GenBank database. After screening single samples with conserved primers, we confirmed the positivity rates of CoVs in each province (Figure 1).

### Complete genome sequencing

We selected samples positive for CoVs that were representative of each province for genomic sequencing. The initial results revealed that they belonged to the genus *Alphacoronavirus* and showed close relationships with BtCoVHKU8, 1A, or 1B. We therefore amplified the cDNA using degenerate primers designed by multiple alignment of the genomes of BtCoVHKU8 (NC010438), BtCoV1A (NC010437), and BtCoV1B (NC010436). Based on the genetic sequences obtained, sequence-specific primers were used in the subsequent PCR amplifications. The primers used to amplify the fragments of each virus are available upon request. The 5′/3′ ends of the viral genomes were confirmed by rapid amplification of cDNA ends (RACE) using a 5′ RACE kit (Invitrogen) and 3′ RACE kit (TaKaRa, Japan). For PCRs with weak or non-specific products, the desired DNA fragments were cloned in DNA vectors (pGEM-T Easy vector; Promega, USA). Multiple clones from a PCR were selected for standard DNA sequencing. Sequences were assembled and edited manually to produce the final viral genome sequences. Each full genome was deduced from a single specimen.

### Sequencing complete *RdRp* and *S* genes

Some positive samples did not undergo complete genome sequencing because of limited amounts of sample. To increase the accuracy of subsequent phylogenetic analyses, we amplified the complete *RdRp* genes of four strains and the complete *S* genes of three strains, in addition to the complete genomes of six strains. Sequencing was performed using the primers available from the genomic sequencing, as previously described. The sequences of the PCR products were assembled manually to produce the complete *RdRp* and *S* gene sequences.

## Genomic analysis

The nucleotide (nt) sequences of the genomes and the deduced amino acid (aa) sequences of the ORFs were predicted using Vector NTI software (Invitrogen) or the ORF Finder tool of NCBI (http://www.ncbi.nlm.nih.gov/gorf/gorf.html). Pairwise genome sequence alignment was conducted with EMBOSS Needle software (www.ebi.ac.uk/Tools/psa/emboss_needle/) using the default parameters. MEGA5.0 (Tamura et al., 2011) was used to align nt and deduced aa sequences with the MUSCLE package and default parameters. The best substitution model was then evaluated using the Model Selection package implemented in MEGA5. Phylogenetic analyses were processed by the maximum-likelihood method with an appropriate model, to create phylogenetic trees with 1,000 bootstrap replicates (Guindon et al., 2010). Protein-family analysis was performed with PFAM (Bateman et al., 2002) and InterProScan (Apweiler et al., 2001). Predictions of transmembrane domains were performed with TMHMM (Sonnhammer et al., 1998).

## Recombination analysis

Recombinations among five genomes were detected with SimPlot software (version 3.5.1). We used a sliding window of 1,000 nt, which moved in steps of 300 nt, and applied the Genetic Algorithms for Recombination Detection program in the DataMonkey software package (http://www.datamonkey.org) (Kosakovsky Pond et al., 2006). When multiple breakpoints were detected between the non-recombinant and recombinant models, they were assessed by comparing the corrected Akaike's Information Criterion scores. The Kishino-Hasegawa test was applied to verify if the adjacent sequence fragments yielded significant topological incongruence.

## Nucleotide sequence accession numbers

All genome sequences have been submitted to GenBank. The accession numbers for the bat alpha-CoVs are KJ473795 to KJ473805.

**Compliance and ethics** *The author(s) declare that they have no conflict of interest.*

**Acknowledgements** *This study was supported by the Program for Changjiang Scholars and Innovative Research Team in University of China (IRT13007), the National S&T Major Project "China Mega-Project for Infectious Disease" (2011ZX10004-001, 2014ZX10004001) from China, the National Natural Science Foundation of China (81501773), and the PUMC Youth Fund and Fundamental Research Funds for the Central Universities (3332015095, 3332015006).*

Apweiler, R., Attwood, T.K., Bairoch, A., Bateman, A., Birney, E., Biswas, M., Bucher, P., Cerutti, L., Corpet, F., Croning, M.D., Durbin, R., Falquet, L., Fleischmann, W., Gouzy, J., Hermjakob, H., Hulo, N., Jonassen, I., Kahn, D., Kanapin, A., Karavidopoulou, Y., Lopez, R., Marx, B., Mulder, N.J., Oinn, T.M., Pagni, M., Servant, F., Sigrist, C.J., and Zdobnov, E.M. (2001). The InterPro database, an integrated documentation resource for protein families, domains and functional sites. Nucleic Acids Res 29, 37–40.

Bateman, A., Birney, E., Cerruti, L., Durbin, R., Etwiller, L., Eddy, S.R., Griffiths-Jones, S., Howe, K.L., Marshall, M., and Sonnhammer, E.L. (2002). The Pfam protein families database. Nucleic Acids Res 30, 276–280.

Bosch, B.J., van der Zee, R., de Haan, C.A., and Rottier, P.J. (2003). The coronavirus spike protein is a class I virus fusion protein: structural and functional characterization of the fusion core complex. J Virol 77, 8801–8811.

Chen, L.L., Ou, H.Y., Zhang, R., and Zhang, C.T. (2003). ZCURVE_CoV: a new system to recognize protein coding genes in coronavirus genomes, and its applications in analyzing SARS-CoV genomes. Biochem Biophys Res Commun 307, 382–388.

Chu, D.K., Peiris, J.S., Chen, H., Guan, Y., and Poon, L.L. (2008). Genomic characterizations of bat coronaviruses (1A, 1B and HKU8) and evidence for co-infections in *Miniopterus* bats. J Gen Virol 89, 1282–1287.

Cui, J., Han, N., Streicker, D., Li, G., Tang, X., Shi, Z., Hu, Z., Zhao, G., Fontanet, A., Guan, Y., Wang, L., Jones, G., Field, H.E., Daszak, P., and Zhang, S. (2007). Evolutionary relationships between bat coronaviruses and their hosts. Emerg Infect Dis 13, 1526–1532.

King, A.M.Q., Adams, M.J., Carstens, E.B. (2011). Virus Taxonomy, Classification and Nomenclature of Viruses. Ninth Report of the International Committee on Taxonomy of Viruses, International Union of Microbiological Societies, Virology Division. London: Elsevier Academic Press, 806–828.

Dveksler, G.S., Pensiero, M.N., Cardellichio, C.B., Williams, R.K., Jiang, G.S., Holmes, K.V., and Dieffenbach, C.W. (1991). Cloning of the mouse hepatitis virus (MHV) receptor: expression in humaaan and hamster cell lines confers susceptibility to MHV. J Virol 65, 6881–6891.

Gonzalez, J.M., Gomez-Puertas, P., Cavanagh, D., Gorbalenya, A.E., and Enjuanes, L. (2003). A comparative sequence analysis to revise the current taxonomy of the family *Coronaviridae*. Arch Virol 148, 2207–2235.

Graham, R.L., and Baric, R.S. (2010). Recombination, reservoirs, and the modular spike: mechanisms of coronavirus cross-species transmission. J Virol 84, 3134–3146.

Guindon, S., Dufayard, J.F., Lefort, V., Anisimova, M., Hordijk, W., and Gascuel, O. (2010). New algorithms and methods to estimate maximum-likelihood phylogenies: assessing the performance of PhyML 3.0. Syst Biol 59, 307–321.

Herrewegh, A.A., Smeenk, I., Horzinek, M.C., Rottier, P.J., and de Groot, R.J. (1998). Feline coronavirus type II strains 79-1683 and 79-1146 originate from a double recombination between feline coronavirus type I and canine coronavirus. J Virol 72, 4508–4514.

Holmes, K.V., and Enjuanes, L. (2003). Virology. The SARS coronavirus: a postgenomic era. Science 300, 1377–1378.

Jonassen, C.M., Kofstad, T., Larsen, I.L., Lovland, A., Handeland, K., Follestad, A., and Lillehaug, A. (2005). Molecular identification and characterization of novel coronaviruses infecting graylag geese (*Anser anser*), feral pigeons (*Columbia livia*) and mallards (*Anas platyrhynchos*). J Gen Virol 86, 1597–1607.

Kosakovsky Pond, S.L., Posada, D., Gravenor, M.B., Woelk, C.H., and Frost, S.D. (2006). GARD: a genetic algorithm for recombination detection. Bioinformatics 22, 3096–3098.

Lai, M.M. (1990). Coronavirus: organization, replication and expression of genome. Annu Rev Microbiol 44, 303–333.

Lai, M.M., and Cavanagh, D. (1997). The molecular biology of coronaviruses. Adv Virus Res 48, 1–100.

Lai, M.M.C., and Holmes, K.V. (2001). Coronaviruses. In: Knipe, D.M., Howley, P.M., Griffin, D.E., Lamb, R.A., Martin, M.A., Roizman, B., and Straus, S.E., eds. Fields Virology. Philadelphia: Lippincott Williams & Wilkins 1163–1185.

Lau, S.K., Li, K.S., Tsang, A.K., Shek, C.T., Wang, M., Choi, G.K., Guo, R., Wong, B.H., Poon, R.W., Lam, C.S., Wang, S.Y., Fan, R.Y., Chan, K.H., Zheng, B.J., Woo, P.C., and Yuen, K.Y. (2012a). Recent transmission of a novel alphacoronavirus, bat coronavirus HKU10, from Leschenault's rousettes to pomona leaf-nosed bats: first evidence of interspecies transmission of coronavirus between bats of different suborders. J Virol 86, 11906–11918.

Lau, S.K., Woo, P.C., Li, K.S., Huang, Y., Tsoi, H.W., Wong, B.H., Wong, S.S., Leung, S.Y., Chan, K.H., and Yuen, K.Y. (2005). Severe acute respiratory syndrome coronavirus-like virus in Chinese horseshoe bats. Proc Natl Acad Sci USA 102, 14040–14045.

Lau, S.K., Woo, P.C., Li, K.S., Huang, Y., Wang, M., Lam, C.S., Xu, H., Guo, R., Chan, K.H., Zheng, B.J., and Yuen, K.Y. (2007). Complete genome sequence of bat coronavirus HKU2 from Chinese horseshoe bats revealed a much smaller spike gene with a different evolutionary lineage from the rest of the genome. Virology 367, 428–439.

Lau, S.K., Woo, P.C., Yip, C.C., Fan, R.Y., Huang, Y., Wang, M., Guo, R., Lam, C.S., Tsang, A.K., Lai, K.K., Chan, K.H., Che, X.Y., Zheng, B.J., and Yuen, K.Y. (2012b). Isolation and characterization of a novel Betacoronavirus subgroup A coronavirus, rabbit coronavirus HKU14, from domestic rabbits. J Virol 86, 5481–5496.

Li, W., Shi, Z., Yu, M., Ren, W., Smith, C., Epstein, J.H., Wang, H., Crameri, G., Hu, Z., Zhang, H., Zhang, J., McEachern, J., Field, H., Daszak, P., Eaton, B.T., Zhang, S., and Wang, L.F. (2005). Bats are natural reservoirs of SARS-like coronaviruses. Science 310, 676–679.

Liu, S., Chen, J., Kong, X., Shao, Y., Han, Z., Feng, L., Cai, X., Gu, S., and Liu, M. (2005). Isolation of avian infectious bronchitis coronavirus from domestic peafowl (*Pavo cristatus*) and teal (*Anas*). J Gen Virol 86, 719–725.

Makino, S., Keck, J.G., Stohlman, S.A., and Lai, M.M. (1986). High-frequency RNA recombination of murine coronaviruses. J Virol 57, 729–737.

Miller-Butterworth, C.M., Jacobs, D.S., and Harley, E.H. (2003). Strong population substructure is correlated with morphology and ecology in a migratory bat. Nature 424, 187–191.

Rest, J.S., and Mindell, D.P. (2003). SARS associated coronavirus has a recombinant polymerase and coronaviruses have a history of host-shifting. Infect Genet Evol 3, 219–225.

Shirato, K., Maeda, K., Tsuda, S., Suzuki, K., Watanabe, S., Shimoda, H., Ueda, N., Iha, K., Taniguchi, S., Kyuwa, S., Endoh, D., Matsuyama, S., Kurane, I., Saijo, M., Morikawa, S., Yoshikawa, Y., Akashi, H., and Mizutani, T. (2012). Detection of bat coronaviruses from *Miniopterus fuliginosus* in Japan. Virus Genes 44, 40–44.

Song, H.D., Tu, C.C., Zhang, G.W., Wang, S.Y., Zheng, K., Lei, L.C., Chen, Q.X., Gao, Y.W., Zhou, H.Q., Xiang, H., Zheng, H.J., Chern, S.W., Cheng, F., Pan, C.M., Xuan, H., Chen, S.J., Luo, H.M., Zhou, D.H., Liu, Y.F., He, J.F., Qin, P.Z., Li, L.H., Ren, Y.Q., Liang, W.J., Yu, Y.D., Anderson, L., Wang, M., Xu, R.H., Wu, X.W., Zheng, H.Y., Chen, J.D., Liang, G., Gao, Y., Liao, M., Fang, L., Jiang, L.Y., Li, H., Chen, F., Di, B., He, L.J., Lin, J.Y., Tong, S., Kong, X., Du, L., Hao, P., Tang, H., Bernini, A., Yu, X.J., Spiga, O., Guo, Z.M., Pan, H.Y., He, W.Z., Manuguerra, J.C., Fontanet, A., Danchin, A., Niccolai, N., Li, Y.X., Wu, C.I., and Zhao, G.P. (2005). Cross-host evolution of severe acute respiratory syndrome coronavirus in palm civet and human. Proc Natl Acad Sci USA 102, 2430–2435.

Sonnhammer, E.L., von Heijne, G., and Krogh, A. (1998). A hidden Markov model for predicting transmembrane helices in protein sequences. Proc Int Conf Intell Syst Mol Biol 6, 175–182.

Tamura, K., Peterson, D., Peterson, N., Stecher, G., Nei, M., and Kumar, S. (2011). MEGA5: molecular evolutionary genetics analysis using maximum likelihood, evolutionary distance, and maximum parsimony methods. Mol Biol Evol 28, 2731–2739.

Tang, X.C., Zhang, J.X., Zhang, S.Y., Wang, P., Fan, X.H., Li, L.F., Li, G., Dong, B.Q., Liu, W., Cheung, C.L., Xu, K.M., Song, W.J., Vijaykrishna, D., Poon, L.L., Peiris, J.S., Smith, G.J., Chen, H., and Guan, Y. (2006). Prevalence and genetic diversity of coronaviruses in bats from China. J Virol 80, 7481–7490.

van Boheemen, S., de Graaf, M., Lauber, C., Bestebroer, T.M., Raj, V.S., Zaki, A.M., Osterhaus, A.D., Haagmans, B.L., Gorbalenya, A.E., Snijder, E.J., and Fouchier, R.A. (2012). Genomic characterization of a newly discovered coronavirus associated with acute respiratory distress syndrome in humans. MBio doi: 10.1128/mBio.00473-12.

Vijaykrishna, D., Smith, G.J., Zhang, J.X., Peiris, J.S., Chen, H., and Guan, Y. (2007). Evolutionary insights into the ecology of coronaviruses. J Virol 81, 4012–4020.

Weiss, S.R., and Navas-Martin, S. (2005). Coronavirus pathogenesis and the emerging pathogen severe acute respiratory syndrome coronavirus. Microbiol Mol Biol Rev 69, 635–664.

Woo, P.C., Lau, S.K., Lam, C.S., Lai, K.K., Huang, Y., Lee, P., Luk, G.S., Dyrting, K.C., Chan, K.H., and Yuen, K.Y. (2009). Comparative analysis of complete genome sequences of three avian coronaviruses reveals a novel group 3c coronavirus. J Virol 83, 908–917.

Woo, P.C., Lau, S.K., Lam, C.S., Lau, C.C., Tsang, A.K., Lau, J.H., Bai, R., Teng, J.L., Tsang, C.C., Wang, M., Zheng, B.J., Chan, K.H., and Yuen, K.Y. (2012). Discovery of seven novel Mammalian and avian coronaviruses in the genus deltacoronavirus supports bat coronaviruses as the gene source of alphacoronavirus and betacoronavirus and avian coronaviruses as the gene source of gammacoronavirus and deltacoronavirus. J Virol 86, 3995–4008.

Woo, P.C., Lau, S.K., Li, K.S., Poon, R.W., Wong, B.H., Tsoi, H.W., Yip, B.C., Huang, Y., Chan, K.H., and Yuen, K.Y. (2006a). Molecular diversity of coronaviruses in bats. Virology 351, 180–187.

Woo, P.C., Lau, S.K., Yip, C.C., Huang, Y., Tsoi, H.W., Chan, K.H., and Yuen, K.Y. (2006b). Comparative analysis of 22 coronavirus HKU1 genomes reveals a novel genotype and evidence of natural recombination in coronavirus HKU1. J Virol 80, 7136–7145.

Woo, P.C., Lau, S.K., and Yuen, K.Y. (2006c). Infectious diseases emerging from Chinese wet-markets: zoonotic origins of severe respiratory viral infections. Curr Opin Infect Dis 19, 401–407.

Woo, P.C., Lau, S.K., Huang, Y., and Yuen, K.Y. (2009). Coronavirus diversity, phylogeny and interspecies jumping. Exp Biol Med 234, 1117–1127.

Woo, P.C., Lau, S.K., and Yuen, K.Y. (2006). Infectious diseases emerging from Chinese wet-markets: zoonotic origins of severe respiratory viral infections. Curr Opin Infect Dis 19, 401–407.

Wu, Z., Yang, L., Ren, X., He, G., Zhang, J., Yang, J., Qian, Z., Dong, J., Sun, L., Zhu, Y., Du, J., Yang, F., Zhang, S., and Jin, Q. (2015). Deciphering the bat virome catalog to better understand the ecological diversity of bat viruses and the bat origin of emerging infectious diseases. ISME J 10, 609–620.

Zeng, Q., Langereis, M.A., van Vliet, A.L., Huizinga, E.G., and de Groot, R.J. (2008). Structure of coronavirus hemagglutinin-esterase offers insight into corona and influenza virus evolution. Proc Natl Acad Sci USA 105, 9065–9069.

**Open Access** This article is distributed under the terms of the Creative Commons Attribution License which permits any use, distribution, and reproduction in any medium, provided the original author(s) and source are credited.
